# Supplementary material for: Interobserver variability in interim PET assessment in Hodgkin lymphoma—reasons and solutions
Source: PLoS One. 2023 Mar 28;18(3):e0283694. doi: 10.1371/journal.pone.0283694 (PMC10047538; doi:10.1371/journal.pone.0283694)
Supplement: S1 Table — (PDF) [file pone.0283694.s001.pdf]

Supporting information

S1 Table. Comparison of the quantitative Deauville score (qDS) in major visual discrepancies

Major visual discrepancies			
Discrepancy between	N	Concordant qDS	Ratio
vDS 1 and 3	23	-	-
vDS 2 and 4	6	0	0%
vDS 3 and 5	3	0	0%
vDS 1 and 4	15	-	-
vDS 2 and 5	1	0	0%
vDS 1 and 5	0	-	-
Overall	48	0	0%
